# Fatty acids, membrane viscosity, serotonin and ischemic heart disease

**DOI:** 10.1186/1476-511X-9-97

**Published:** 2010-09-08

**Authors:** Massimo Cocchi, Lucio Tonello, Giovanni Lercker

**Affiliations:** 1 Institute "Paolo Sotgiu" Quantitative and Evolutionary Psychiatry and Cardiology, L.U.de.S. University, Via dei Faggi 4 - Quartiere La Sguancia -Lugano-Pazzallo 6900, Switzerland; 2 DIMORFIPA, University of Bologna, Via Tolara di Sopra 50-Ozzano dell'Emilia 40064-Bologna, Italy; 3 DISA, University of Bologna, Viale Fanin 44 - Bologna 40127, Italy

## Abstract

Novel markers for ischemic heart disease are under investigation by the scientific community at international level.

This work focuses on a specific platelet membrane fatty acid condition of viscosity which is linked to molecular aspects such as serotonin and G proteins, factors involved in vascular biology.

A suggestive hypothesis is considered about the possibility to use platelet membrane viscosity, in relation to serotonin or, indirectly, the fatty acid profile, as indicator of ischemic risk.

## Background

Because of peculiar characteristic of platelet in depression and in ischemic heart disease [1-4], we have first investigated the platelet fatty acid profile in 50 subjects with diagnosis of ischemic heart disease, 60 subjects in health clinic conditions, 84 subjects with clinical diagnosis of Major Depression [5-7].

The fatty acids values of the 3 populations have been administered to a Self Organizing Map (SOM), mixing healthy and pathological individuals and hidding the information on to their own pathological condition. As a result, the SOM, after having identified the fatty acids markers (Palmitic, Linoleic and Arachidonic for depression and Oleic, Linoleic and Arachidonic for ischemia), was able to map the three populations (depressive vs normal and ischemic vs normal) using the specific fatty acids, recognizing as similar those belonging to the same population and, in the meanwhile, different those belonging to one population from the other ones [5-7].

After the experiment, to classify other groups of subjects, the platelet fatty acid pattern has been analyzed in 80 subjects with Morphea, in 31 subjects with Scleroderma and in 45 young people, in healthy clinical conditions, for a total of 350 subjects.

## Concept of chemical-physical membrane state

In the case of biological membranes we use the terms of "fluidity", "stiffness," permeability "functionality", and "stickiness", related or connected with biological effects of considerable importance.

Fluidity and viscosity are two terms used in physics with specific meaning: the viscosity is a dynamic property of matter and is defined as the skid resistance of two fluid layers between them, in a real system treated as a package of fluid layers superimposed (in slow linear motion), which can vary with temperature for the same molecules, while the fluidity is the opposite.

Rigidity, permeability and function are, however, characteristics of the membrane and are terms used to describe a physical and biological membrane behavior on the physiology of the cell. The viscosity of the membrane, of course, is related to the composition of fatty acids constituting the lipid bilayer (membrane folds).

With reference to the membrane folds, they are usually very close and the distance may be very small in the case of saturated fatty acids, distance which tends to increase, replacing these with unsaturated fatty acids, much more as they are unsaturated.

## Membrane viscosity and serotonin

It has been demonstrated that the membrane viscosity is a peculiar characteristic in depressive subjects [7,8] and that regulates serotonin receptors uptake [9].

Among all its function serotonin is also involved in some aspects of cardiac events such as coronary artery disease, in aggregation and vasoconstriction [10,11].

Platelet takes up serotonin from plasma by the serotonin transporters and it is, then, secreted by the platelet dense granules during platelet activation, playing a role in platelet aggregation and vasoconstriction of surrounding blood vessels. Recent studies suggest that intracellular serotonin may also play a role in platelet activation through covalent linkage to small G proteins, activating G protein signaling pathways and stimulating platelet aggregation [12].

Total serotonin levels and the number of platelets have been found significantly higher among patients whith coronary artery disease [11].

## Hypothesis

Independently of the SOM results, among all the fatty acid profiles, three fatty acids (Palmitic, Linoleic and Arachidonic), unexpectedly, had a constant total amount (53. 33 ± 3.43) representing the larger amount of platelet membrane fatty acids in all cases studied.

Being the Palmitic Acid opposed to Linoleic Acid and to Arachidonic Acid (Table 1), according to the saturation and unsaturation characteristics, it has been possible to calculate a coefficient, called B3 (Table 2), which gives an indication of viscosity, using the following formula:

**Table 1:** Description of the fatty acids considered

i	Name	
1	Palmitic Acid	C 16:0

2	Linoleic Acid	C 18:2

3	Arachidonic Acid	C 20:4

**Table 2:** Value of the B3 index in the subjects studied

Fatty acid	MP/Mwt	Normal subjects (average ± SD)	B3 index	Depressive (average ± SD)	B3 index	Ischemic (average ± SD)	B3 index
C 16:0	0.246	20.68 ± 2.15	5.089	17.92 ± 4.462	4.408	23.32 ± 3.17	5.737

C 18:2 n6	-0.018	19.40 ± 2.69	-0.348	16.71 ± 3.359	-0,301	10.51 ± 3.44	-0.189

C 20:4 n6	-0.164	14.06 ± 2.41	-2.306	19.03 ± 3.839	-3.121	15.17 ± 3.01	-2.488

			**2,434**		**0.986**		**3.060**

$${\text{B}}_{\text{2}}=\sum_{\text{i}=\text{1}}^{\text{3}}{\text{(A}}_{\text{i}}\frac{{\text{MeltingPoint}}_{\text{i}}}{{\text{MolecularWeight}}_{\text{i}}}\text{)}$$

Where:

Ai = percentage of i-th Fatty Acid

mwi = molecular weight of i-th Fatty Acid

mpi = melting point of i-th Fatty Acid (°C)

The result is consistent with the knowledge that correlates platelet membrane viscosity and function, in the conditions investigated (normal, ischemic and depressive subjects).

This could lead to the idea of a new ischemic risk assessment, considering each fatty acid concentration, within the same total percentage.

## Implication of the hypothesis

As shown in table 2 the B3 index is significantly higher (about 30%) in ischemic than in normal subjects and 3 times higer than in depressive subjects.

For the properties that link membrane viscosity to the platelet serotonin receptor uptake and for the role of serotonin in coronary artery disease, the evaluation of the chemical-physical characteristic should be utilized to forecast the ischemic risk, in agreement with the experimental results obtained in ischemic heart disease subjects through the SOM use [6,7].

Recent studies have, also, pointed out alterations of endocannabinoids (arachidonate derivatives) in human platelets during various human pathologies and in relation to serotonin and depression [13-18]. The information contained in these works seems relevant to the reported data and support the proposed hypothesis.

## Conclusion

In our opinion, studies on gene expression and molecular aspects of platelets, in ischemic heart disease, should take into account the membrane fatty acids ratio and its viscosity.

## Competing interests

The authors declare that they have no competing interests.

## Authors' contributions

MC and LT were particularly involved in data collection and data analysis. GL was involved to calculate the B3 index. All authors were involved in the interpretation of the data.

All the authors have been involved in drafting and revising the manuscript and have read and approved the final manuscript.

## References

[B1] Nelson GJ Schmidt PS Bartolini GL Kelley DS Kyle D The effect of dietary arachidonic acid on platelet function, platelet fatty acid composition, and blood coagulation in humans Lipids 1997 32 421 5 10.1007/s11745-997-0055-7 9113631

[B2] Randon NJ Gandhi C Siafaka-Kapadai A Olson MS Hanahan DJ The inhibition of platelet-activating factor-induced platelet activation by oleic acid is associated with a decrease in polyphosphoinositide metabolism J Pharmacol Exp Ther 1997 281 1264 1271 2170407

[B3] Takahashi S Reduction of blood platelet serotonin levels in manic and depressed patients Folia Psychiatr Neurol Jpn 1976 30 475 86 1021543 10.1111/j.1440-1819.1976.tb02670.x

[B4] Kim HL Plaisant O Leboyer M Gay C Kamal L Devynck MA Reduction of platelet serotonin in major depression (endogenous depression) C R Seances Acad Sci III 1982 295 619 22 6819060

[B5] Cocchi M Tonello L Platelets, Fatty Acids, Depression and Cardiovascular Ischemic Pathology Progress in Nutrition 2007 9 94 104

[B6] Cocchi M Tonello L Tsaluchidu S Puri BK The use of self-organizing maps to study fatty acids in neuropsychiatric disorders BMC Psychiatry 2008 8 S3 10.1186/1471-244X-8-S1-S3 18433513 PMC2330078

[B7] Cocchi M Tonello L Bio molecular considerations in Major Depression and Ischemic Cardiovascular Disease Central Nervous System Agents in Medicinal Chemistry 2010 10 97 107 20518726 10.2174/187152410791196378

[B8] Tonello L Cocchi M The Cell Membrane: Is it a Bridge from Psychiatry to Quantum Consciousness? NeuroQuantology 2010 8 54 60

[B9] Heron DS Shinitzkyt M Hershkowitz M Samuel D Lipid fluidity markedly modulates the binding of serotonin to mouse brain membranes Proc Natl Acad Sci 1980 77 7463 7467 10.1073/pnas.77.12.7463 6938985 PMC350524

[B10] Berger M Gray JA Roth BL The Expanded Biology of Serotonin Annu Rev Med 2009 60 355 66 10.1146/annurev.med.60.042307.110802 19630576 PMC5864293

[B11] Vikenes K Farstad M Nordrehaug JE Serotonin Is Associated with Coronary Artery Disease and Cardiac Events Circulation 1999 100 483 489 10430761 10.1161/01.cir.100.5.483

[B12] Walther DJ Peter JU Winter S Höltje M Paulmann N Grohmann M Vowinckel J Alamo-Bethencourt V Wilhelm CS Ahnert-Hilger G Bader M Serotonylation of small GTPases is a signal transduction pathway that triggers platelet alpha-granule release Cell 2003 62 115 851 10.1016/s0092-8674(03)01014-6 14697203

[B13] Maccarrone M Rossi S Bari M De Chiara V Fezza F Musella A Gasperi V Prosperetti C Bernardi G Finazzi-Agrò A Cravatt BF Centonze D Anandamide inhibits metabolism and physiological actions of 2-arachidonoylglycerol in the striatum Nat Neurosci 2008 11 152 159 10.1038/nn2042 18204441

[B14] Rossi C Pini LA Cupini ML Calabresi P Sarchielli P Endocannabinoids in platelets of chronic migraine patients and medication-overuse headache patients: relation with serotonin levels Eur J Clin Pharmacol 2008 64 1 8 10.1007/s00228-007-0391-4 18004553

[B15] Macarrone M Bari M Menichelli A Giuliani E Del Principe D Finazzi-Agro A Human platelets bind and degrade 2-arachidonoylglycerol, which activates these cells through a cannabinoid receptor Eur J Biochem 2001 268 819 825 10.1046/j.1432-1327.2001.01942.x 11168423

[B16] Maccarrone M Bari M Principe DD Finazzi-Agro A Activation of human platelets by 2-arachidonoylglycerol is enhanced by serotonin Thromb Haemost 2003 89 340 347 12574815

[B17] Cupini LM Bari M Battista N Argiro G Finazzi-Agro A Calabresi P Maccarrone M Biochemical changes in endocannabinoid system are expressed in platelets of female but not male migraineurs Cephalalgia 2006 26 277 281 10.1111/j.1468-2982.2005.01031.x 16472333

[B18] Bari M Battista N Fezza F Gasperi V Maccarrone M New insights into endocannabinoid degradation and its therapeutic potential Mini Rev Med Chem 2006 6 257 268 10.2174/138955706776073466 16515464

